# Resolutions of Respect to Dr. Theodore F. Chupein

**Published:** 1901-10

**Authors:** 


### RESOLUTIONS OF RESPECT TO DR. THEODORE F. CHUPEIN.

At a regular meeting of the Pennsylvania Association of Dental Surgeons, held Tuesday evening, April 9, 1901, the Committee on Resolutions upon the death of Dr. Theodore F. Chupein submitted the following, which were accepted and adopted:

Whereas, With profound regret the Pennsylvania Association of Dental Surgeons is called upon to notice the death of Dr. Theodore F. Chupein, an old, tried, and faithful member, it is meet and fitting that it should place on record its appreciation of his long and faithful services as a member, and of his far-reaching, earnest, and valued services to the profession he loved.

Dr. Chupein became a member of the Association September 13, 1876, and at once, with earnestness, took and continued to take with unflagging zeal an active part in all its work. He was elected recording secretary October 9, 1877, and by re-election continued to serve until his death, March 23, 1901.

His earnestness in professional work, his faithfulness as a member and officer of this Association, his manliness, and his friendliness well merit our most profound appreciation and respect. Be it therefore

*Resolved,* That by the death of Dr. Theodore F. Chupein the dental profession has lost an earnest and progressive member, and this society a firm and fast friend.

*Resolved,* That, bowing in humble submission to the will of Him who doeth all things well, we hereby express our heartfelt sympathy to his bereaved wife and family; and be it further

*Resolved,* That a copy of these resolutions be transmitted to his family, and published in the dental journals.

Wilbur F. Litch,
William H. Trueman,
*Committee.*

J. Clarence Salvas,
*Secretary.*

